# A Longitudinal Study of Eating Rituals in Patients With Anorexia Nervosa

**DOI:** 10.3389/fpsyg.2019.00015

**Published:** 2019-01-18

**Authors:** Simona Calugi, Elisa Chignola, Riccardo Dalle Grave

**Affiliations:** Department of Eating and Weight Disorders, Villa Garda Hospital, Garda, Italy

**Keywords:** eating rituals, anorexia nervosa, eating disorder psychopathology, cognitive behavioral therapy, inpatient

## Abstract

**Background:** Eating rituals are any problematic behaviors involving food. They are usually observed in patients with anorexia nervosa, but research into these behaviors and their role in treatment outcomes is lacking.

**Objective:** We set out to assess the presence of eating rituals in patients with anorexia nervosa treated by means of intensive enhanced cognitive behavioral therapy (ICBT-E), in addition to their change over time and role as potential predictors of treatment outcome.

**Materials and Methods:** Ninety adult female inpatients with anorexia nervosa were recruited. The Participants’ body mass index (BMI), and scores for Starvation Symptoms Inventory (SSI), Eating Disorder Examination (EDE), and Brief Symptom Inventory (BSI) were recorded, and a purpose-designed 9-item checklist of eating rituals was completed by trained dieticians during assisted eating – an integral part of the ICBT-E. The Structured Clinical Interview for DSM-IV was used at admission to identify the presence of coexisting axis I psychiatric disorders. All other tests were administered at baseline (admission), the end of treatment and 6-month follow-up. BMI, EDE, and BSI were also re-administered after 4 weeks of treatment in order to examine how refeeding affects these variables.

**Results:** We found a correlation at baseline between eating rituals and both general and eating-disorder psychopathology scores. Eating rituals were also associated with the presence of at least one comorbid anxiety disorder. ICBT-E treatment was associated with a significant reduction in eating rituals, as well as a significant increase in BMI and improved eating-disorder and general psychopathology. However, our most relevant finding was that neither baseline eating ritual scores nor their change during treatment was associated with either BMI or general or eating-disorder psychopathology scores taken at either the end of therapy or at 6-month follow-up.

**Conclusion:** Neither the presence of nor change in eating rituals influence treatment outcomes in patients with anorexia nervosa.

## Introduction

Eating rituals are any problematic behaviors around food, its preparation, its consumption, or any situation involving food or eating (Herpertz-Dahlmann, 2009). Examples are food being cut into very small pieces, separated on the plate, being chewed a certain number of times, excessively chewed before swallowing, and eaten food group by food group. Other rituals include the meticulous measurement or arrangement of food.

Ritualized eating behaviors were first described in the Minnesota Starvation Experiment, which accurately reported the effects of dietary restriction and underweight in 36 young male volunteers without eating-disorder psychopathology. For example, one participant of the study reported the strange and interesting processes the men developed for eating the little food that was provided: “*… eating became a ritual... Some people diluted their food with water to make it seem like more. Others would put each little bite and hold it in their mouth a long time to savor it. So eating took a long time*” (Keys et al., 1950). Subsequently, some clinicians described ritualized eating behaviors in patients with anorexia nervosa (Mazure et al., 1994; Herpertz-Dahlmann, 2009; Tchanturia et al., 2013), and the fifth edition of the Diagnostic and Statistical Manual of Mental Disorders (DSM-5) recently clarified that obsessive-compulsive disorder should not better explained by the symptoms of another mental disorder, such as the ritualized eating behaviors seen in eating disorders (American Psychiatric Association, 2013).

Some authors have hypothesized that eating rituals could contribute to the onset and maintenance of eating disorders, and that, after weight normalization and reduction of eating disorder psychopathology, the risk of relapse may increase over time in patients who continue to engage in ritualized behaviors around eating (Sunday and Halmi, 2000; Bellace et al., 2012). However, according to enhanced cognitive behavioral therapy for eating disorders (CBT-E), the inordinately slow and ritualized eating seen in some underweight patients does not usually need to be addressed, as it is an expression of undereating and underweight and is therefore reversed as patients regain weight and improve the eating-disorder psychopathology (Fairburn et al., 2003). That being said, we have not yet seen any empirical investigation into the role of eating rituals in treatment outcomes or the way in which they change in patients with anorexia nervosa receiving a specialist treatment. Nevertheless, such an investigation would shed interesting light on whether or not there is, in fact, an association between such behaviors and aspects of and improvements in eating-disorder psychopathology. Therefore, our primary aim was to evaluate whether eating rituals are predictors of BMI or general or eating-disorder psychopathology scores recorded at complete weight restoration and/or 6-month follow-up. Indeed, if eating rituals are in fact predictors of treatment outcome, this could affect the choice of clinical procedures during therapy. The secondary aims of the study were to assess the proportion of eating rituals displayed and the change in eating rituals and eating-disorder and general psychopathology scores after 4 weeks of refeeding and at the end of treatment in a sample of patients with anorexia nervosa receiving ICBT-E – an intensive treatment based on CBT-E.

## Materials and Methods

### Study Design

The study was prospective longitudinal and included four assessment time-points.

### Setting

The study was conducted exclusively at Villa Garda Hospital Eating Disorder Unit (Verona, Italy) in patients voluntary receiving an adapted version of CBT-E (Dalle Grave, 2012), known as ICBT-E, for anorexia nervosa. Participants were recruited from March 2010 through January 2013. Baseline data were collected at admission to the Villa Garda Hospital Unit. After 4 weeks of treatment (corresponding to the refeeding period), patients completed self-report questionnaires. Outcome data were gathered 20 weeks from admission, when the participants finished the inpatient treatment and, finally, 6 months after discharge.

### Participants

Ninety consecutively treated adult female patients were recruited for this study. They were between 18 and 65 years of age, and all met the DSM-5 diagnostic criteria for anorexia nervosa (American Psychiatric Association, 2013). Diagnosis was made by the referring clinician and confirmed by an eating disorder specialist (R.D.G.) by means of the validated Italian version of the 16th edition of the Eating Disorder Examination (EDE) interview (Fairburn et al., 2008; Calugi et al., 2015). Patients with daily substance use and acute psychotic disorders were excluded.

At 6-month follow-up, patients were contacted by phone and invited to the Villa Garda Hospital Unit to complete the follow-up assessment and have their body weight recorded.

### Treatment

The treatment has three main goals: (i) to remove the eating-disorder psychopathology; (ii) to correct the mechanisms that have been maintaining this psychopathology; and (iii) to ensure that the changes achieved are lasting. The program consists of 13 weeks of inpatient treatment and 7 weeks of therapy in day hospital. In total, patients receive 20 weeks of CBT-E sessions, delivered both individually and in groups, as well as dietician-assisted eating in the first 4 weeks. Patients receive dietician-assisted eating and progressive increases in the daily energy content of their diet from 1500 to 2500 kcal until they reach a BMI of 18.5 kg/m^2^. The goal is to achieve a steady weight gain of 1/1.5 kg per week. As recommended by CBT-E, the specifically trained dieticians do not directly address the patient’s eating rituals during assisted eating, and their role in this phase is to help patients to eat using cognitive behavioral procedures (e.g., eating without being influenced by internal signals of hunger or fullness; eating without being influenced by preoccupations about eating or food; using the self-monitoring record in real time). Indeed, in the initial phase of ICBT-E, the focus is on refeeding and mitigating eating concerns; the overvaluation of shape, weight, and their control – considered the core psychopathology of anorexia nervosa – is instead addressed from 4 weeks on, as the patients begin to achieve weight restoration. Once a patient reaches a BMI of 19.0 kg/m^2^, their daily calorie intake is adjusted to enable them to maintain a stable body weight within a 3-kg range of this target. In this phase patients continue to address their individual eating-disorder psychopathology. Further information on the treatment received by participants can been found in the ICBT-E manual (Dalle Grave, 2012).

### Source of Bias

We used several procedures to minimize bias in the study. During the study design we evaluated the inter-rater reliability of the instrument used to assess eating rituals; moreover, the clinical experts who measured outcomes were different to those attending the patient during hospitalization. To avoid transfer bias, we tried to minimize patient loss to follow-up by offering convenient office hours and a free examination by a physician. Finally, the inclusion of confounding baseline variables in the statistical models was aimed at reducing the role of confounders during data analysis.

### Ethics Statement

This study was carried out in accordance with the recommendations of by the Villa Garda Hospital Institutional Review Board. As per the Declaration of Helsinki, all subjects gave written informed consent. The protocol was approved by the Villa Garda Hospital Institutional Review Board (N° Prog. 329 CEP).

### Assessment

Body mass index (BMI) and scores for Starvation Symptoms Inventory (SSI), Eating Disorder Examination (EDE), and Brief Symptom Inventory (BSI) were acquired at baseline (admission to the Unit), and again at the end of treatment, and after 6 months of follow-up. BMI, BSI and SSI questionnaires were also re-administered after 4 weeks of treatment in order to assess whether refeeding had any effect on these variables. A 9-item checklist of eating rituals was completed by the assisting dieticians during assisted eating before and after treatment and at 4 weeks, and the Structured Clinical Interview for DSM-IV was used at baseline to identify any coexisting axis I psychiatric disorders.

#### Body Weight and Body Mass Index

Participants’ height was measured using a wall-mounted stadiometer (Wall-Mounted Mechanical Height Rod Model 00051A; Wunder). They were weighed in only their underwear using a beam balance scale (Seca Digital Wheelchair Scale Model 664), and their BMI was then calculated as their body weight in kilograms divided by their height in square meters.

#### Eating Rituals

A checklist to measure the presence/absence of eating rituals was developed by the two assisting dieticians. It included the ritualized eating behaviors recorded by the Minnesota Starvation Experiment (Keys et al., 1950) and those observed during ICBT-E assisted eating in patients with anorexia nervosa. A final version of nine items was used to evaluate the presence of eating rituals during the meals given the participants in this study, namely: mixing food continuously; disassembling food; counting morsels; cutting food into geometric shapes; cutting food into small pieces; chewing for a long time; taking long breaks; eating slowly; hiding food; and throwing food on the floor. At baseline, after 4 weeks, and at the end of treatment, assisting dieticians observed each participant during lunch and completed the checklist, indicating the presence/absence of each behavior. The global score was obtained by summing the number of eating rituals displayed by each participant. A random sample of 35 participants was selected and scored by both dieticians independently; the inter-rater reliability was very high (*rho* = 0.92).

#### Starvation Symptoms

The Starvation Symptom Inventory (SSI) (Calugi et al., 2017), was designed to measure the symptoms of starvation in patients with eating disorder who are underweight via 15 self-assessment items. Items evaluate the starvation symptoms reported in the Minnesota Starvation Experiment (Keys et al., 1950) as well as those detected by clinical observation. Specifically, participants were asked to estimate how many days out of the 4 weeks (28 days) preceding the interview that they experienced the itemized symptoms. Scores are provided on a Likert-type scale on which ‘never’ is scored 0 and ‘always’ as 6. Good internal consistency (alpha = 0.91), test–retest reliability (*r* = 0.90), and convergent and criterion validity have previously been reported for the global SSI score (Calugi et al., 2017). The SSI showed high internal consistency (alpha = 0.92) in the present sample.

#### Eating Disorder Features

The validated Italian version of the 16th edition of the EDE interview (Fairburn et al., 2008; Calugi et al., 2015) was administered at admission, the end of treatment and after 6 months of follow-up to assess eating disorder features. Our sample showed high internal consistency (alpha = 0.91) for the EDE global score.

#### General Psychiatric Features

The Brief Symptom Inventory (BSI), validated Italian version (De Leo et al., 1993), was used to identify clinical symptoms that indicate emotional distress. The BSI comprises three scales for measuring global psychological distress, and nine subscales for assessing specific aspects of distress (Derogatis and Melisaratos, 1983). For the purposes of our study we recorded the Global Severity Index (GSI) and the Anxiety and Obsession-Compulsion subscale scores. The BSI-GSI displayed high internal consistency (alpha = 0.97) in our sample. In addition, the Structured Clinical Interview for DSM-IV (First et al., 1995) was used at baseline to identify the presence of coexisting axis I psychiatric disorders.

### Statistical Analyses

All statistical analyses were carried out using SPSS software (IBM SPSS Statistics, version 23.0). Continuous variables were categorized as means (SD), and categorical variables as frequencies and percentages. In order to evaluate the relationships between the mean number of eating rituals displayed and the demographic and baseline clinical characteristics of the sample, Spearman’s correlation coefficients were calculated. The effects of ICBT-E on each outcome (BMI, and eating ritual, global SSI global EDE, and EDE subscale scores) were assessed using repeated measures analysis of variance. Both intention-to-treat (using last observation carried forward) and completers analyses were carried-out. Linear regression analyses were performed in order to evaluate whether eating rituals at baseline, their change after 4 weeks, and/or at the end of therapy, could predict treatment outcomes (BMI, EDE, BSI, and SSI scores, weight restoration) at the end of therapy and/or at 6 months after discharge. Data are presented for completers. Baseline BMI, and general and eating-disorder psychopathology scores were included in the models as confounding variables in order to prevent baseline severity of BMI, eating disorder and general psychopathology affecting the relationship between eating rituals and outcome measures.

## Results

### Participant Descriptors

The mean age of the 90 female participants was 26.3 years (*SD* = 8.3), and their mean baseline BMI was 14.4 kg/m^2^ (*SD* = 1.7). According to DSM-5 criteria, 63 participants (70%) were diagnosed with anorexia nervosa restricting type and 27 (30%) with anorexia nervosa binge-eating/purging type. Their mean age at anorexia nervosa onset was 17.4 years (*SD* = 5.2), and they reported a mean duration of illness of 8.8 years (*SD* = 7.5), and mean weight suppression (highest body weight minus current body weight) of 16.4 kg (*SD* = 11.1). Seventy-five patients (83.3%) were single, while 2 (2.2%) were divorced or separated. Forty-one (45.5%) were in full-time education, and 26 (28.9%) were employed. Many participants (*n* = 78, 86.7%) had recently undertaken an outpatient treatment for eating disorders without success, while 37 (41.1%) had previously received psychoactive medication and 85 (94.4%) some form of psychotherapy.

The Structured Clinical Interview for DSM-IV (First et al., 1995) revealed the following axis I comorbidities: current major depressive episode in 45.6%, an obsessive compulsive disorder in 10%, a post-traumatic stress disorder in 11.1%, a panic disorder with or without agoraphobia in 7.8%, a specific or social phobia in 14.4%, and a generalized anxiety disorder in 4.4%; globally, 25% of the sample displayed comorbidity with at least one anxiety disorder.

The mean number of baseline eating rituals was 3.8 (*SD* = 2.2), median 4; 11 (12.2%) participants displayed no eating rituals and one patient displayed 8 out of the 9 on the checklist. The correlation coefficients between eating rituals and demographic and clinical features at admission are shown in Table 1. Eating rituals were significantly but weakly correlated with age and eating-disorder (except EDE Eating Concern subscale) and general psychopathology, but not with weight suppression, BMI, duration of illness, eating disorder behaviors, BSI Anxiety or Obsession-Compulsion subscale scores, or starvation symptoms (SSI scores).

**Table 1:** Spearman correlations between eating rituals and clinical characteristics in 90 female patients with anorexia nervosa at baseline.

	Eating rituals
Age, years	-0.24^∗^
Duration of eating disorder, years	-0.20
Body mass index (kg/m^2^)	-0.08
Weight suppression (kg)	0.13
**Eating-disorder psychopathology (EDE)**	
Overall severity	0.27^∗∗^
Dietary restraint	0.26^∗^
Eating concern	0.17
Weight concern	0.25^∗^
Shape concern	0.23^∗^
**Eating disorder behavior (EDE)**	
Objective binge-eating episodes	-0.07
Self-induced vomiting	-0.04
Laxative misuse	0.01
Excessive exercise	0.05
**Brief symptom inventory**	
Global severity index	0.35^∗∗^
Anxiety	-0.01
Obsession-compulsion	0.01
**Starvation symptom inventory**	0.19


*EDE, Eating Disorder Examination; weight suppression = highest body weight – current body weight; ^∗^*p* < 0.05; ^∗∗^*p* < 0.001.*

A comparison of the eating rituals in participants with and without a comorbid axis I disorder indicated that the only difference was between those with at least one comorbid anxiety disorder and those without and was that the former presented a higher mean number of baseline eating rituals (4.7 ± 1.9 vs. 3.4 ± 2.2, *z* = 0.021). All other comparisons were found to be not significant. Moreover, a comparison between patients with restricting and binge-eating/purging types of anorexia nervosa indicated rates of eating rituals that were not significantly different (4.0 ± 2.1 vs. 3.2 ± 2.2, respectively, *z* = 0.121).

### Drop-Out Rates

The program was completed by seventy-three patients (81.1%), whereas 17 (18.9%) left the program before the end. Eight (8/90, 8.9%) dropped out during the first 4 weeks, and 9 (9/90, 10%) thereafter. We found no significant differences in baseline demographic or clinical features between drop-outs and completers (all *p’*s > 0.05). Sixty-two completers (62/73, 84.9%) had 6-month follow-up data available. There were no significant differences in baseline variables between completers were assessed after 6 months and those who did not attend the follow-up appointment.

### Treatment Outcome

Both intent-to-treat and completers analyses indicated significant improvements in all variables measured during the treatment and at follow-up (Table 2). In detail, BMI, EDE Dietary Restraint subscale, SSI and BSI global scores significantly improved from baseline to the end of therapy. Among completers, a large percentage of patients (75.3%, 55/73) achieved weight restoration (BMI ≥ 18.5 kg/m^2^). However, from the end of treatment to 6-month follow-up, a significant worsening was observed in these variables and only 25/62 patients (40.3%) had a BMI ≥ 18.5 kg/m^2^. In contrast, EDE global, Eating Concern, Weight Concern and Shape Concern subscale scores and BSI Anxiety and Obsession-Compulsion subscale scores showed and maintained a significant improvement from baseline to the end of treatment and at 6-month follow-up. Finally, eating rituals improved from admission to 4 weeks and to the end of treatment, and the percentage of participants with at least one eating ritual at baseline who completed the treatment decreased from 87.7 to 71.2 and 41.1% after 4 weeks and at the end of treatment, respectively (*p* < 0.001).

**Table 2:** Intention-to-treat and completers analysis at all time points.

	INTENTION-TO-TREAT ANALYSIS (*n* = 90)					
	
	Baseline^a^	Four weeks^b^	End of treatment^c^	6-Month follow-up^d^	*p*-value	Bonferroni correction
**Body mass index (kg/m^2^)**	14.4 (1.7)	15.6 (1.7)	18.2 (2.3)	17.3 (2.7)	<0.001	a < b,c,d
						b < c,d
						c > d
**Eating disorder examination**						
Global score	3.6 (1.4)	-	2.1 (1.4)	2.3 (1.5)	<0.001	a < c,d
Dietary restraint	4.0 (1.6)	-	1.9 (1.6)	2.4 (1.8)	<0.001	a < c,d
						c < d
Eating concern	3.3 (1.7)	-	1.5 (1.5)	1.8 (1.6)	<0.001	a < c,d
Weight concern	3.2 (1.8)	-	2.2 (1.5)	2.2 (1.7)	<0.001	a < c,d
Shape concern	3.7 (1.6)	-	2.8 (1.5)	2.7 (1.7)	<0.001	a < c,d
**Starvation symptom inventory**	55.1 (21.1)	35.6 (20.5)	24.2 (17.6)	30.2 (21.9)	<0.001	a < b,c,d
						b < c
						c > d
**Brief symptom inventory**						
Global severity index	1.9 (0.9)	1.4 (0.8)	0.9 (0.7)	1.1 (0.8)	<0.001	a > b,c,d
						b > c,d
						c < d
Anxiety	2.3 (1.1)	1.8 (1.1)	1.3 (1.0)	1.5 (1.1)	<0.001	a > b,c,d
						b > c,d
Obsession-compulsion	2.1 (1.1)	1.6 (1.2)	1.2 (1.0)	1.3 (1.1)	<0.001	a > b,c,d
						b > c,d
**Eating disorder rituals**	3.8 (2.2)	2.5 (2.2)	1.4 (1.8)	-	<0.001	a > b,c
						b > c

**COMPLETERS ANALYSIS**						

**Body mass index (kg/m^2^)**	14.5 (1.8)	15.8 (1.6)	19.0 (1.22)	17.7 (2.6)	<0.001	a < b,c,d
						b < c,d
						c > d
**Eating disorder examination**						
Global score	3.7 (1.4)	-	1.8 (1.1)	2.0 (1.4)	<0.001	a > c,d
Dietary restraint	4.1 (1.6)	-	1.4 (1.1)	2.1 (1.7)	<0.001	a<c,d
						c < d
Eating concern	3.4 (1.5)	-	1.1 (1.0)	1.6 (1.4)	<0.001	a < c,d
Weight concern	3.4 (1.9)	-	2.0 (1.4)	1.9 (1.6)	<0.001	a < c,d
Shape concern	3.8 (1.6)	-	2.5 (1.5)	2.3 (1.8)	<0.001	a < c,d
**Starvation symptoms inventory**	55.6 (20.3)	35.9 (20.4)	20.6 (14.1)	29.0 (22.0)	<0.001	a > b,c,d
						b > c
						c < d
**Brief symptoms inventory**						
Global severity index	1.9 (0.9)	1.4 (0.8)	0.8 (0.6)	1.1 (0.8)	<0.001	a > b,c,d
						b > c,d
						c < d
Anxiety	2.2 (1.0)	1.7 (1.1)	1.1 (0.8)	1.4 (1.0)	<0.001	a > b,c,d
						b > c
Obsession-compulsion	2.0 (1.0)	1.5 (1.2)	0.9 (0.8)	1.2 (1.0)	<0.001	a > c,d
						b > c
**Eating rituals**	3.7 (2.2)	2.4 (2.2)	1.1 (1.5)	-	<0.001	a > b,c
						b > c


*Repeated measure analysis of variance. a = Baseline; b = Four weeks; c = End of treatment; d = 6-Month follow-up.*

### Eating Rituals, Their Change and Treatment Outcome

Stepwise linear regression analysis including baseline eating rituals as the independent variable and BMI, EDE, and BSI subscale scores, and global SSI score measured at the end of treatment (and at 6-month follow-up, in another model) as dependent variables, controlling for baseline BMI, and global EDE and BSI scores, indicated that eating rituals were not significantly associated with outcome variables measured at end of treatment (adjusted R square = 0.10; R square change = 0.13) or at 6-month follow-up (adjusted R square = 0.10; R square change = 0.13) (all *p’s* > 0.05). No outcome variable at the end of therapy was significantly associated with the change in eating rituals from baseline to 4 weeks of treatment (adjusted R square = 0.08; R square change = 0.12). Similarly, no outcome variable at 6-month follow-up was significantly associated with the change in eating rituals from baseline to 4 weeks of treatment (adjusted R square = 0.06; R square change = 0.11) or with eating rituals at the end of treatment (adjusted R square = -0.03; R square change = 0.02). Moreover, in a subsequent linear regression model, eating rituals did not predict the achievement of weight restoration at either the end of therapy or at 6-month follow-up.

## Discussion

This study, which aimed to assess the presence of eating rituals, their change during inpatient treatment, and their role in treatment outcome in 90 adult female patients treated with ICBT-E, yielded three main findings. First, eating rituals appear to be associated with age, and general and eating-disorder psychopathology scores, but not at all with baseline weight suppression, BMI, duration of illness or eating disorder behaviors. Despite numerous clinical observations, to our knowledge this is the first study to detect a significant correlation between the presence of eating rituals and younger age and higher rates of general and eating-disorder psychopathology scores in patients with anorexia nervosa. Moreover, we found that the presence of at least one comorbid anxiety disorder was associated with a higher number of eating rituals. These results, if confirmed and expanded upon by future research, indicate that eating rituals represent a clinical expression of eating disorder psychopathology, are not associated with either the severity of underweight or the amount of weight lost, but are exacerbated by the co-existence of an anxiety disorder.

The second finding is that the number of eating rituals, like BMI, eating disorder and general psychopathology, were significantly reduced after the first 4 weeks of ICBT-E treatment, and continued to decrease until the end of therapy. These data confirm the effectiveness of ICBT-E in an inpatient setting (Dalle Grave et al., 2013) and indicate that the improvement in eating rituals begins during refeeding, and continues until the end of treatment.

The third, and most important, finding was that the baseline presence of eating rituals, their change after the first 4 weeks of treatment and their presence at the end of treatment were not significant predictors of BMI or eating disorder or general psychopathology at either the end of treatment or at 6-month follow-up. Moreover, achieving weight restoration was not associated with eating rituals at baseline, 4 weeks or the end of therapy. If confirmed, these data support the CBT-E strategy of not directly addressing eating rituals during the treatment, because only rarely do they represent an obstacle to treatment or maintain the eating disorder psychopathology mechanisms (Fairburn et al., 2003).

Our findings may be considered robust, as the patient cohort was both sizable and representative – being recruited from an inpatient unit affiliated with the Italian National Health System. Furthermore, to evaluate the eating rituals, we used a simple checklist compiled by dieticians expert in eating disorders during assisted eating. Moreover, we assessed eating rituals in both at the end of treatment and in its early stages and this enabled us to evaluate early changes in these behaviors.

However, it should be noted that even though the inter-rater reliability of the checklist we used to assess eating rituals was very high (*rho* = 0.92), it has not yet been validated. As there is no well-validated instrument to measure eating rituals, we cannot guarantee that all the most relevant behaviors were included. However, the instrument was developed by dieticians with considerable experience of observing patients with eating disorders during meals, which could mitigate this bias. Moreover, the sample included only adult females, voluntarily treated in an inpatient setting, and it is therefore possible that our findings are not applicable to adolescents, males or outpatients. Finally, we recruited no control group, which limits the significance our results. However, it should be noted that this remains a major unresolved challenge for the field of eating disorders, as it is very difficult to recruit a suitable control group of underweight subjects without eating disorder psychopathology.

## Conclusion

The present study is the first to assess the role of eating rituals behaviors in the outcomes of a treatment for anorexia nervosa. The data suggest that neither the presence of ritualized eating behaviors nor their change affect treatment outcomes. If confirmed, these findings indicate that there is no the need to target eating rituals directly in patients with anorexia nervosa, thereby confirming CBT-E recommendations. However, with a view to clarifying the relationship between anxiety disorder and eating rituals, and to confirm the role of eating rituals in maintaining eating disorder psychopathology and in treatment outcomes in other populations (i.e., adolescents or outpatients), future research should be aimed at developing good, validated instruments to assess eating rituals in patients with eating disorders.

## Author Contributions

All authors contributed to and approved the final version of the manuscript. Specifically, SC contributed to conducting study concept and design, protocol development, and data collection, and was responsible for statistical analyses and interpretation of results, as well as drafting and finalizing the manuscript. EC contributed to data collection, results interpretation, and drafting and finalizing the manuscript. RD supervised the study concept and design development, as well as protocol development and data collection, interpretation of results, and drafting and finalizing the manuscript.

## Conflict of Interest Statement

The authors declare that the research was conducted in the absence of any commercial or financial relationships that could be construed as a potential conflict of interest. The reviewer MG declared a past co-authorship with several of the authors SC, EC, and RD to the handling Editor.
